# From Cell Death to Metabolism: Holin-Antiholin Homologues with New Functions

**DOI:** 10.1128/mBio.01963-17

**Published:** 2017-12-05

**Authors:** Marielle H. van den Esker, Ákos T. Kovács, Oscar P. Kuipers

**Affiliations:** a Department of Molecular Genetics, Groningen Biomolecular Sciences and Biotechnology Institute, University of Groningen, Groningen, The Netherlands; b Bacterial Interactions and Evolution Group, Department of Biotechnology and Biomedicine, Technical University of Denmark, Kongens Lyngby, Denmark

**Keywords:** *Bacillus subtilis*, *Staphylococcus aureus*, antiholin, evolution, holin, metabolism, programmed cell death, pyruvate

## Abstract

Programmed cell death in bacteria is generally triggered by membrane proteins with functions analogous to those of bacteriophage holins: they disrupt the membrane potential, whereas antiholins antagonize this process. The holin-like class of proteins is present in all three domains of life, but their functions can be different, depending on the species. Using a series of biochemical and genetic approaches, in a recent article in *mBio*, Charbonnier et al. (mBio 8:e00976-17, 2017, https://doi.org/10.1128/mBio.00976-17) demonstrate that the antiholin homologue in *Bacillus subtilis* transports pyruvate and is regulated in an unconventional way by its substrate molecule. Here, we discuss the connection between cell death and metabolism in various bacteria carrying genes encoding these holin-antiholin analogues and place the recent study by Charbonnier et al. in an evolutionary context.

## COMMENTARY

Bacteria are unicellular organisms long thought to lack programmed cell death (PCD), as this phenomenon seemed hard to justify evolutionarily at the individual cell level. However, in the past decades, it has become clear that bacteria can commit “suicide” and actively kill themselves by following a genetic cell death program under certain conditions. One example includes biofilm formation of *Staphylococcus aureus*, where a small subpopulation lyses to provide DNA that glues the extracellular matrix together. This process is regulated by the Cid/Lrg network, a genetic program that regulates cell death in a manner similar to eukaryotic apoptosis (1). The Cid/Lrg network integrates signals related to the metabolic status of the cell and environmental circumstances via two regulators, LytSR and CidR. These regulatory proteins balance transcription of the antiholin-like gene *lrgA* and the holin-like gene *cidA*, which encode proteins located in the cell wall that can permeabilize the cell wall, resulting in lysis. Overflow metabolism, and specifically the balance between acetate and acetoin formation in *S. aureus*, determines the fate of the cell, revealing a link between PCD and metabolism. Homologues of components of the Cid/Lrg network have been identified in a wide range of other bacteria, both Gram-negative and Gram-positive bacteria, indicating that PCD is widely conserved (2).

The Gram-positive model bacterium *Bacillus subtilis* contains genes encoding homologues of these genes in its genome, annotated as *ywbH* (*cidA* homologue) and *ysbA* (*lrgA* homologue). We have recently demonstrated that YsbA and its two-component regulatory system (TCS) LytST do not play a role in programmed cell death of *B. subtilis* but instead have a metabolic function and are involved in pyruvate utilization (3). An elegant study of Charbonnier and coworkers published in a recent article in *mBio* (4) has further elaborated on the functions of these proteins and revealed that the *ysbAB* operon encodes a hetero-oligomeric membrane complex that acts as a facilitated pyruvate transporter. The operon was therefore renamed *pftAB* (named *pft* for pyruvate facilitated transporter).

The transcriptional regulation of the *pftAB* operon suggests a role for *pftAB* in pyruvate metabolism. On the one hand, CcpA, the master regulator of the carbon catabolite response (CCR), inhibits the expression of *pftAB* in the presence of glucose and malate, the preferred carbon sources of *B. subtilis*, by binding to the −35 region of the promoter. The CCR is principally mediated via the regulation of pyruvate transport, and not by modulating intracellular enzyme expression, thereby efficiently balancing pyruvate levels in the presence of extracellular pyruvate. This energy-efficient strategy is used more often by microorganisms and particularly by *B. subtilis*, e.g., the CcpA-mediated repression of the uptake transporter of arabinose, galactose, and xylose, *araE* in *B. subtilis* (5). On the other hand, the TCS LytST induces the expression of *pftAB*. The signal that LytS senses has not yet been experimentally elucidated, but it could respond to extracellular pyruvate levels or uptake fluxes. LytT subsequently activates *pftAB* transcription in the presence of pyruvate by binding to its promoter region.

Pyruvate is a key intermediate in various metabolic pathways, and LytT is likely required for balancing intracellular pyruvate levels, thereby influencing the metabolic state of the cell. The peculiarity of the LytST system as described by Charbonnier and colleagues (4) resides in its multiple regulatory functions, instead of just its traditional feed-forward mechanism. The authors show that *pftAB* induction by LytT increases up to 1 mM extracellular pyruvate, but under excess pyruvate conditions, *pftAB* transcription is retroinhibited via LytST. Interestingly, *pftAB* inhibition can also be observed in the presence of malate. After uptake, malate is converted to pyruvate by malic enzymes, resulting in increased intracellular pyruvate levels and an ensuing decrease of LytT-dependent *pftAB* activation. Apparently, LytT activation is influenced in various ways, be it direct (e.g., via pyruvate or another nutrient) or indirect (e.g., degradation or dephosphorylation by another regulatory protein). Nonetheless, membrane-embedded LytS could also have multiple functions or another function than the intuitively expected function. Via LytST, the bacterial cells seem to precisely sense the accessible pyruvate and adjust its uptake accordingly.

Although the study of Charbonnier et al. (4) reports that no other genes are directly induced by LytT in the presence of pyruvate, a heuristic microarray study of Kobayashi et al. (6) revealed that *pftAB* was induced by LytT, while *ywbH* appeared to be repressed. The function of YwbH and its regulation by LytT have not yet been experimentally explored, but YwbH is currently annotated as a putative holin-like protein based on its homology to the prolytic protein CidA in *S. aureus*. So far, no connection to PCD has been found, but *ywbH* is expressed at high levels in the presence of malate (3). Hence, repression by LytT, either directly or indirectly, might suggest a metabolic function of this gene that could potentially expand our knowledge on the pyruvate/malate metabolic pathway and its homeostasis.

**FIG 1:**
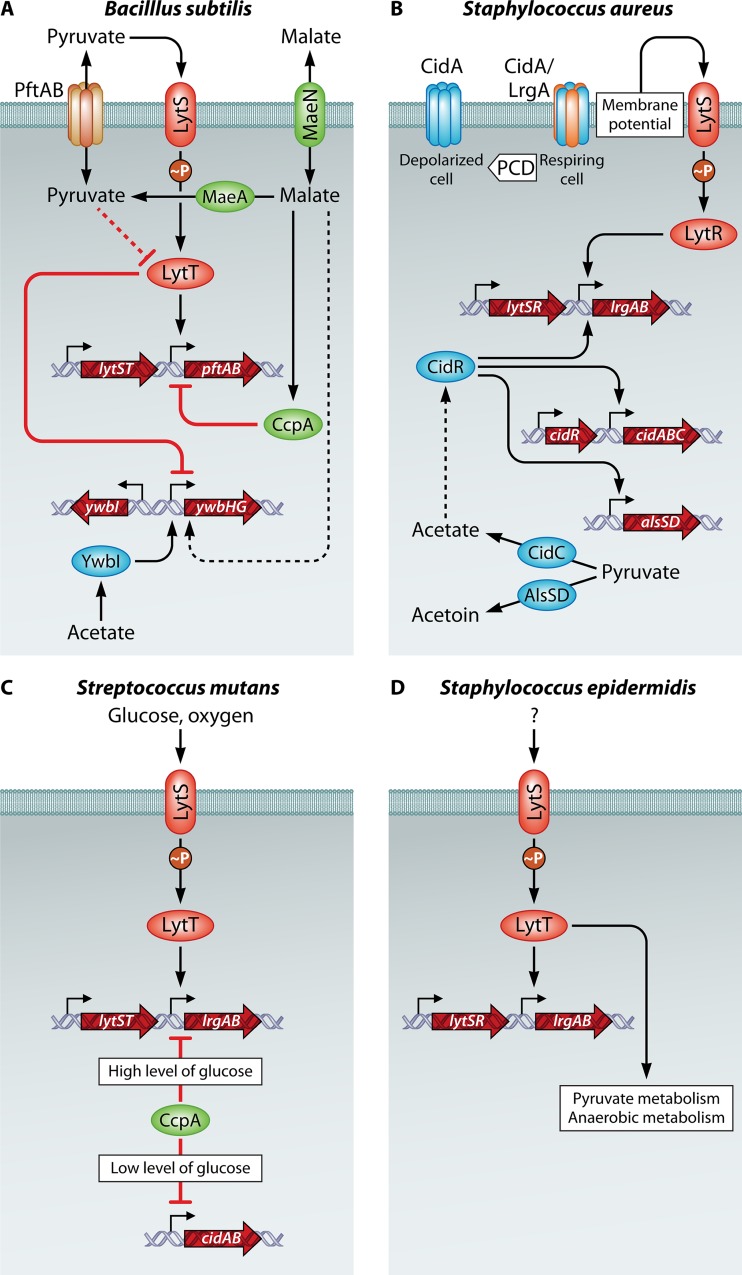
Schematic representation of the regulatory pathways in *B. subtilis*, *S. aureus*, *S. mutans*, and *S. epidermidis*. Arrows depict transcriptional activation, metabolic conversion, or transport, while red T-bars indicate negative regulation. Direct and indirect interactions are indicated by solid and dashed lines, respectively.

LrgA and LytSR (or LytST) are present in various organisms, but only a few studies have been conducted that elucidate their direct role, among which the function of LytSR has been best characterized in *S. aureus* (2). LytS responds to changes in the membrane potential: upon dissipation, LytR induces expression of the antiholin-like protein LrgA, and thereby, the cell attempts to prevent total membrane permeabilization. In other organisms, the deletion of *lytSR* revealed distinct roles for this regulatory pathway: in *Streptococcus mutans*, LytST governs *lrgAB* expression in response to glucose and reactive oxygen species (ROS), while in *Staphylococcus epidermidis*, LytSR regulates cell death during biofilm formation and pyruvate utilization (7, 8). Thus, it appears that LytSR, although widely conserved, has variable roles in different organisms, all being connected to metabolism or cell death or tentatively both (Fig. 1).

Notably, ubiquitous genes with high homology have diverged considerably in function throughout the course of evolution. However, the link between metabolism, ROS, and PCD appears to be prevalent in nature. In eukaryotes, where apoptosis has been studied in far greater detail, networks regulating metabolism and PCD are deeply intertwined. The metabolic status of a cell determines its fate: whether a cell divides, grows, or dies depends on the environment, nutrient availability, and its energy status. A wide range of sensing mechanisms integrate the diverse signals and control gene expression accordingly. This is especially apparent in the mitochondria, which are thought to have a bacterial origin and where many of the cell’s key metabolic and signaling pathways are routed through. Specifically, the mitochondrial Bcl-2 protein family seems to play an important role in linking metabolism and PCD. This protein family has a dual function in signaling pathways governing metabolism and apoptosis, with certain members having both direct functions in nutrient utilization and apoptosis. In addition, Bcl-2 proteins also indirectly influence apoptosis by altering the metabolic status of the cell (9). The conserved nature of Bcl-2-like proteins suggests that the connection between metabolism and PCD is omnipresent.

The holin and antiholin-like proteins of *S. aureus* have been proposed to be functionally analogous to the Bcl-2 proteins: Bax is a proapoptotic protein causing mitochondrial outer membrane permeabilization and acts similarly to CidA, while the antiapoptotic Bcl-2 protein antagonizes this process analogously to LrgA. In *S. aureus*, CidA is transcribed from the *cidABC* operon and is induced during overflow metabolism in the presence of high pyruvate or acetate levels. The regulatory protein CidR influences both acetate formation by inducing CidC, a pyruvate oxidase, and acetoin formation via AlsSD. The overall balance between acetate and acetoin formation in *S. aureus* makes the difference between life and death, again showing a tie between metabolism and PCD. Certain features of eukaryotic apoptosis could be recognized within bacterial cell death, such as membrane permeabilization and DNA condensation and fragmentation. Furthermore, the metabolic state of a bacterial cell affects its antibiotic susceptibility (10). Therefore, it is plausible that bacteria possess networks with analogous function and metabolic dependencies that influence certain cells within a population to divide, to arrest growth, or when a metabolically unfavorable condition is reached, to lyse.

Although the evolutionary origin of the eukaryotic Bcl-2 protein family is elusive, it has been speculated that Bcl-2 proteins were acquired by gene transfer from the ancient bacterial or viral world (11). Theories related to their origin are inconclusive: some indicate that Bcl-2 homologues have evolved only about 600 to 700 million years ago and were not present in the mitochondrial ancestor (12), while other theories imply the existence of a Bcl-2-like protein in the primordial bacterium that later formed mitochondria and/or chloroplasts (13). The discovery of an orthologue in the plant kingdom that likely evolved from an LrgA-LrgB fusion supports the latter view, as well as the finding that Bcl-2 expression induces bacterial lysis (14, 15). Moreover, the extent to which homologues of *lrgA* and *cidA* are present in bacterial genomes suggests that these genes evolved early in bacterial evolution. *Archaea* also carry genes that encode holin-like proteins, making these proteins, consisting of 58 families, present in all three kingdoms of life (16).

The holin-antiholin class of proteins were originally discovered in bacteriophages, where they modulate host cell lysis during lytic infection (17). A hypothetical model suggests that these proteins could have been acquired by horizontal transfer to an ancient bacterium through integration of these elements into its genome. These ancient bacteria were subsequently acquired by eukaryotes, eventually leading to the appearance of Bcl-2 family proteins in mitochondria and the LrgB class of proteins in plants. While these protein families possibly preserved their function in connection to PCD in diverse species, protein homologues present in other bacteria subsequently diverged in function away from cell death but maintained their connection to metabolism. Alternatively, the involvement in PCD may have evolved independently based on the biochemical features of this membrane protein family. Determining the function of the diverged LrgA/CidA homologues will reveal whether these proteins are truly evolutionarily linked. The thorough investigation of the LrgAB homologue, PftAB, in *B. subtilis* and its association with pyruvate transport via the cell membrane is the first step toward understanding the evolution and functional diversification of this protein family.

## References

[1] Bayles KW 2003 Are the molecular strategies that control apoptosis conserved in bacteria? Trends Microbiol 11:306–311. doi:10.1016/S0966-842X(03)00144-6. 12875813

[2] Bayles KW 2007 The biological role of death and lysis in biofilm development. Nat Rev Microbiol 5:721–726. doi:10.1038/nrmicro1743. 17694072

[3] van den Esker MH, Kovács ÁT, Kuipers OP 2017 YsbA and LytST are essential for pyruvate utilization in *Bacillus subtilis*. Environ Microbiol 19:83–94. doi:10.1111/1462-2920.13454. 27422364

[4] Charbonnier T, Le Coq D, McGovern S, Calabre M, Delumeau O, Aymerich S, Jules M 2017 Molecular and physiological logics of the pyruvate-induced response of a novel transporter in *Bacillus subtilis*. mBio 8:e00976-17. doi:10.1128/mBio.00976-17. 28974613 PMC5626966

[5] Inácio JM, Costa C, de Sá-Nogueira I 2003 Distinct molecular mechanisms involved in carbon catabolite repression of the arabinose regulon in *Bacillus subtilis*. Microbiology 149:2345–2355. doi:10.1099/mic.0.26326-0. 12949161

[6] Kobayashi K, Ogura M, Yamaguchi H, Yoshida K, Ogasawara N, Tanaka T, Fujita Y 2001 Comprehensive DNA microarray analysis of *Bacillus subtilis* two-component regulatory systems. J Bacteriol 183:7365–7370. doi:10.1128/JB.183.24.7365-7370.2001. 11717295 PMC95585

[7] Zhu T, Lou Q, Wu Y, Hu J, Yu F, Qu D 2010 Impact of the *Staphylococcus epidermidis* LytSR two-component regulatory system on murein hydrolase activity, pyruvate utilization and global transcriptional profile. BMC Microbiol 10:287. doi:10.1186/1471-2180-10-287. 21073699 PMC2996381

[8] Ahn S-J, Qu M-D, Roberts E, Burne RA, Rice KC 2012 Identification of the *Streptococcus mutans* LytST two-component regulon reveals its contribution to oxidative stress tolerance. BMC Microbiol 12:187. doi:10.1186/1471-2180-12-187. 22937869 PMC3507848

[9] Andersen JL, Kornbluth S 2013 The tangled circuitry of metabolism and apoptosis. Mol Cell 49:399–410. doi:10.1016/j.molcel.2012.12.026. 23395270 PMC3801185

[10] Lobritz MA, Belenky P, Porter CBM, Gutierrez A, Yang JH, Schwarz EG, Dwyer DJ, Khalil AS, Collins JJ 2015 Antibiotic efficacy is linked to bacterial cellular respiration. Proc Natl Acad Sci U S A 112:8173–8180. doi:10.1073/pnas.1509743112. 26100898 PMC4500273

[11] Aouacheria A, Le Goff E, Godefroy N, Baghdiguian S 2016 Evolution of the BCL-2-regulated apoptotic pathway, p 137–156. *In* Pontarotti P (ed), Evolutionary biology: convergent evolution, evolution of complex traits, concepts and methods. Springer International, Cham, Switzerland.

[12] Aouacheria A, Brunet F, Gouy M 2005 Phylogenomics of life-or-death switches in multicellular animals: Bcl-2, BH3-only, and BNip families of apoptotic regulators. Mol Biol Evol 22:2395–2416. doi:10.1093/molbev/msi234. 16093567

[13] Wang J, Bayles KW 2013 Programmed cell death in plants: lessons from bacteria? Trends Plant Sci 18:133–139. doi:10.1016/j.tplants.2012.09.004. 23083702 PMC3556228

[14] Pang X, Moussa SH, Targy NM, Bose JL, George NM, Gries C, Lopez H, Zhang L, Bayles KW, Young R, Luo X 2011 Active Bax and Bak are functional holins. Genes Dev 25:2278–2290. doi:10.1101/gad.171645.111. 22006182 PMC3219232

[15] Yang Y, Jin H, Chen Y, Lin W, Wang C, Chen Z, Han N, Bian H, Zhu M, Wang J 2012 A chloroplast envelope membrane protein containing a putative LrgB domain related to the control of bacterial death and lysis is required for chloroplast development in *Arabidopsis thaliana*. New Phytol 193:81–95. doi:10.1111/j.1469-8137.2011.03867.x. 21916894

[16] Saier MH, Reddy BL 2015 Holins in bacteria, eukaryotes, and archaea: multifunctional xenologues with potential biotechnological and biomedical applications. J Bacteriol 197:7–17. doi:10.1128/JB.02046-14. 25157079 PMC4288690

[17] Ahn S-J, Rice KC, Oleas J, Bayles KW, Burne RA 2010 The *Streptococcus mutans* Cid and Lrg systems modulate virulence traits in response to multiple environmental signals. Microbiology 156:3136–3147. doi:10.1099/mic.0.039586-0. 20671018 PMC3068699

